# Photoinduced Strain Release and Phase Transition Dynamics of Solid-Supported Ultrathin Vanadium Dioxide

**DOI:** 10.1038/s41598-017-10217-0

**Published:** 2017-08-30

**Authors:** Xing He, Napat Punpongjareorn, Weizheng Liang, Yuan Lin, Chonglin Chen, Allan J. Jacobson, Ding-Shyue Yang

**Affiliations:** 1 Department of Chemistry, University of Houston, Houston, Texas 77204 United States; 2 State Key Laboratory of Electronic Thin Films and Integrated Devices, University of Electronic Science and Technology of China, Chengdu, 610054 China; 3 Department of Physics and Astronomy, University of Texas at San Antonio, San Antonio, Texas 78249 United States; 4 Texas Center for Superconductivity, University of Houston, Houston, Texas 77004 United States

## Abstract

The complex phase transitions of vanadium dioxide (VO_2_) have drawn continual attention for more than five decades. Dynamically, ultrafast electron diffraction (UED) with atomic-scale spatiotemporal resolution has been employed to study the reaction pathway in the photoinduced transition of VO_2_, using bulk and strain-free specimens. Here, we report the UED results from 10-nm-thick crystalline VO_2_ supported on Al_2_O_3_(0001) and examine the influence of surface stress on the photoinduced structural transformation. An ultrafast release of the compressive strain along the surface-normal direction is observed at early times following the photoexcitation, accompanied by faster motions of vanadium dimers that are more complex than simple dilation or bond tilting. Diffraction simulations indicate that the reaction intermediate involved on picosecond times may not be a single state, which implies non-concerted atomic motions on a multidimensional energy landscape. At longer times, a laser fluence multiple times higher than the thermodynamic enthalpy threshold is required for complete conversion from the initial monoclinic structure to the tetragonal lattice. For certain crystalline domains, the structural transformation is not seen even on nanosecond times following an intense photoexcitation. These results signify a time-dependent energy distribution among various degrees of freedom and reveal the nature of and the impact of strain on the photoinduced transition of VO_2_.

## Introduction

Vanadium dioxide (VO_2_) as a classic correlated material continues to attract great attention in various physics, chemistry, and materials science communities, because of its intriguing yet perplexing phase transitions since the first discovery^1^ as well as potential applications of the switching behaviors at around 340 K or below^2–6^. Parameters including temperature, pressure and strain^7^, stoichiometry and doping^8, 9^, structures and morphologies^10^, photoexcitation^11^, and voltage^12^ can all have significant influence on the phases and structures of VO_2_. As a result, the fundamental nature of the material’s insulator-to-metal transition has been a topic of major debate for decades^13–16^, with experiments and theory supporting either a decisive role of anharmonic lattice vibrations^17^, or collaborative Mott-Peierls (or Peierls-Mott) mechanisms emphasizing the dynamical V‒V dimers^18–20^, or principally the Mott physics^21–24^. On the ultrashort time scale, time-resolved optical pump-probe techniques have been employed to scrutinize the intricate interplay between nonequilibrium carriers and ionic motions in order to reveal the underlying physics^25–31^. These wide-ranging results, if taken together, suggest the high sensitivity of VO_2_ to local disorder and inhomogeneity on the nanoscale^32^, which signifies the need of more studies in detail with well-characterized specimen conditions^33^.

From the structural point of view, it is critical to understand, at the atomic level, phase-transition dynamics of a correlated material during the entire transformation process. To date, the reaction path for photoinduced structural phase transition of VO_2_ has been visualized using time-resolved diffraction methods on bulk^34, 35^ and strain-free specimens^35–38^. The general picture is that on the ultrashort time scale of few hundred femtoseconds (fs) or less, dilation of V‒V dimers in the initial low-temperature monoclinic structure (*M*_1_) plays a critical role in the beginning stage of the phase change^11, 34, 37, 39^. Intracell structural^34, 38^ and orbital occupancy^37^ reorganization proceeds on a picosecond (ps) time, followed by lattice transformation to the tetragonal structure (*R*) on the time scale of tens to a hundred ps^34–36, 38^. While this stepwise transformation mechanism—with some differences in the details such as the involvement of the second monoclinic *M*_2_ structure^38^—explains the observed diffraction dynamics, open questions still remain especially when results of the photo- and thermally-induced phase transitions are compared. For instance, a complete photoinduced conversion to the *R* phase was generally presumed at long times (hundreds of ps and later), although the laser fluence required^37^ appears to be many times higher than the energy needed thermally^40^ (the energy threshold question). The percolative nature and nanoscale inhomogeneity of the insulator-to-metal transition in thin films and microcrystals has been observed using near-field nanoimaging techniques^21, 32, 33, 41^, whereas a coherent motion of vanadium ions for all unit cells was generally used, at least for early delay times, in time-resolved diffraction studies^34–38^ (the question about homogeneity of the transition^42, 43^). Furthermore, how lattice strain affects and involves in the phase transition of VO_2_^7, 22, 23^ at ultrashort times has not been well examined. To address these questions with a unified picture, it is necessary to conduct a time-resolved structural study using suitable specimens.

In this contribution, we report the results of ultrafast electron diffraction (UED) made on ultrathin VO_2_ films epitaxially grown on *c*-sapphire, which exhibit moderate out-of-plane compressive and in-plane tensile strains. The good crystallinity yet different epitaxial orientations of the VO_2_ domains allow us to identify the transformation dynamics that take place in different crystallographic directions at different stages of the photoinduced phase transition. Strong evidence is found for non-concerted motions of vanadium ions at ultrashort times, which are conceptually consistent with the observation of nanopuddles and nucleation during the thermal transition^21, 33^. The observed incomplete conversion to the *R* phase at long times also provides additional evidence for the material’s high sensitivity to local environments and agrees with the presence of superheated monoclinic structure(s) and suppression of the structural transition by strain^7, 23, 44^. These results indicate a converging picture for the phase transition of VO_2_ on the nanoscale.

## Results

### Sample characterization and strain analysis

**Figure 1:**
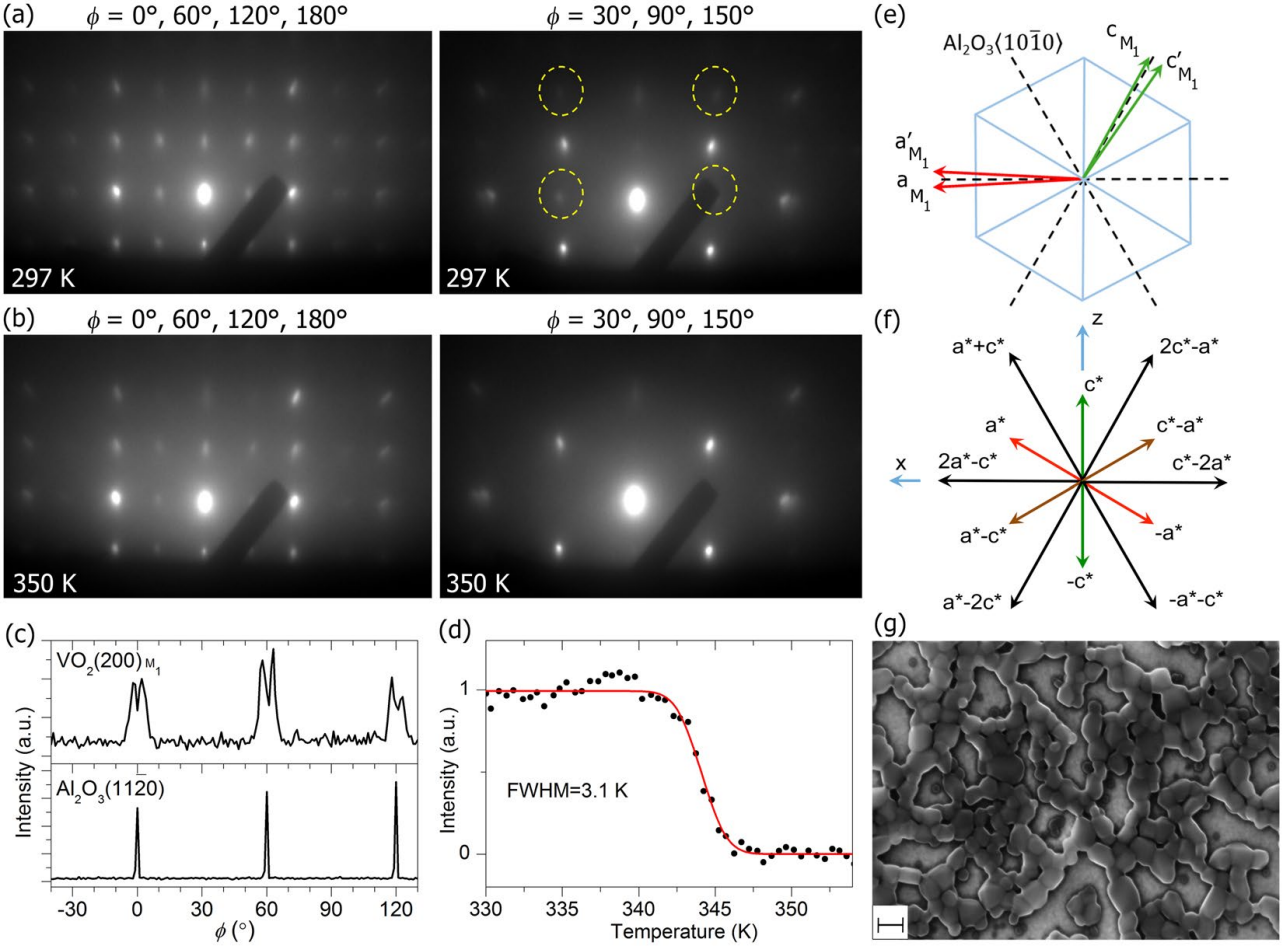
Characterization of 10-nm ultrathin VO_2_ on Al_2_O_3_(0001). (**a**‒**b**) Electron diffraction patterns acquired at selected azimuthal angles (*ϕ*) at 297 K and 350 K, respectively. Dashed circles indicate a few spots that are symmetry-forbidden in the *R* phase. (**c**) X-ray diffraction from a *ϕ* scan showing the epitaxial relationship. The doublet pattern of VO_2_ at ~ ± 2° results from the mismatch between ${\beta }_{{M}_{1}}$ of VO_2_ and the matching angle (60°) of a perfect hexagon. (**d**) Intensity of the *R*-forbidden diffractions as a function of temperature in the heating cycle (black dots). A fit to an error function gives a full-width-at-half-maximum (FWHM) of 3.1 K. (**e**) Epitaxial relationship between VO_2_ (red and green arrows) and *c*-Al_2_O_3_ (blue and dashed lines). The pair of the ${a}_{{M}_{1}}$ and ${c}_{{M}_{1}}$ axes rotates azimuthally and follow the dashed lines. (**f**) Major reciprocal vectors in the ${a}_{{M}_{1}}-{c}_{{M}_{1}}$ plane that are associated with the patterns in a and b; black arrows for *ϕ* = 30°, 90°, 150° and color arrows for *ϕ* = 0°, 60°, 120°, 180°. (**g**) A scanning electron micrograph of the ultrathin specimen used. Scale bar: 200 nm.

The epitaxial VO_2_ ultrathin films were grown on Al_2_O_3_(0001) 10 × 10-mm^2^ substrates by using a polymer-assisted deposition technique^45, 46^. The 10-nm thickness was obtained via control of the viscosity of the precursor solution as well as spin-coating and thermal treatment process. Electron diffraction (ED) and x-ray diffraction (XRD) data show the results of similar patterns for every 60° azimuthal rotation (Fig. 1a‒c), signifying a 6-fold epitaxial relation between VO_2_ and the hexagonal plane of *c*-sapphire with an in-plane mismatch of ~ ± 2° given the ${\beta }_{{M}_{1}}$ angle of VO_2_^45, 47^. We confirm the ${b}_{{M}_{1}}$ axis, not the ${c}_{{M}_{1}}$ axis, to be along the surface normal direction, namely VO_2_(010)//Al_2_O_3_(0001), by comparing the observed ED patterns with simulated ones based on the kinematic scattering theory (see Supplementary Figs. S1‒S2). This assignment is evident compared to that using XRD results only, given the close reciprocal lattice constants of ${b}_{{M}_{1}}^{\ast }$ and ${c}_{{M}_{1}}^{\ast }$ ^47^. Using the sapphire diffractions as references, a careful analysis of the XRD *θ*-2*θ*, *ϕ*, and 2*θ*-*ϕ* scan data indicates a moderate compressive strain of −0.31% in ${b}_{{M}_{1}}$ (VO_2_(020) at 2*θ* = 39.81° and VO_2_(040) at 2*θ* = 89.90°), a tensile strain of + 0.17% ( + 0.35%) in ${a}_{{M}_{1}}$ $({c}_{{M}_{1}})$ (Supplementary Figs. S3‒S4), and a ${\beta }_{{M}_{1}}$ angle of 122.59° that is close to the bulk value^48^. By monitoring the forbidden diffractions for the *R* phase, i.e. those with an odd *h* index from the *M*_1_ phase (Fig. 1a, dashed circles; hereafter referred to as *R*-forbidden diffractions), we obtained a critical transition temperature of *T*_*c*_ = 344 K with a width of 3.1 K in the heating cycle (Fig. 1d), which is consistent with the previous resistivity measurements^45^.

Hence, each ED pattern acquired is the result of simultaneous probing of crystalline domains from different zone axes that are azimuthally rotated by 60°, with the epitaxial relation of VO_2_[100] and VO_2_[001]//Al_2_O_3_ $\langle 10\bar{1}0\rangle$ (dashed lines in Fig. 1e). Such specimen conditions, instead of a randomly-oriented strain-free polycrystalline sample, enable the examination of the effects of surface stress on the VO_2_ dynamics along specific directions (see below). The zone axes that exhibit *R*-forbidden diffractions (Fig. 1a, right panel, and black arrows in Fig. 1f) were used in the experiments of photoinduced dynamics. A scanning electron micrograph shows the average size of crystalline domains to be ~170 nm (Fig. 1g).

### Structural dynamics

**Figure 2:**
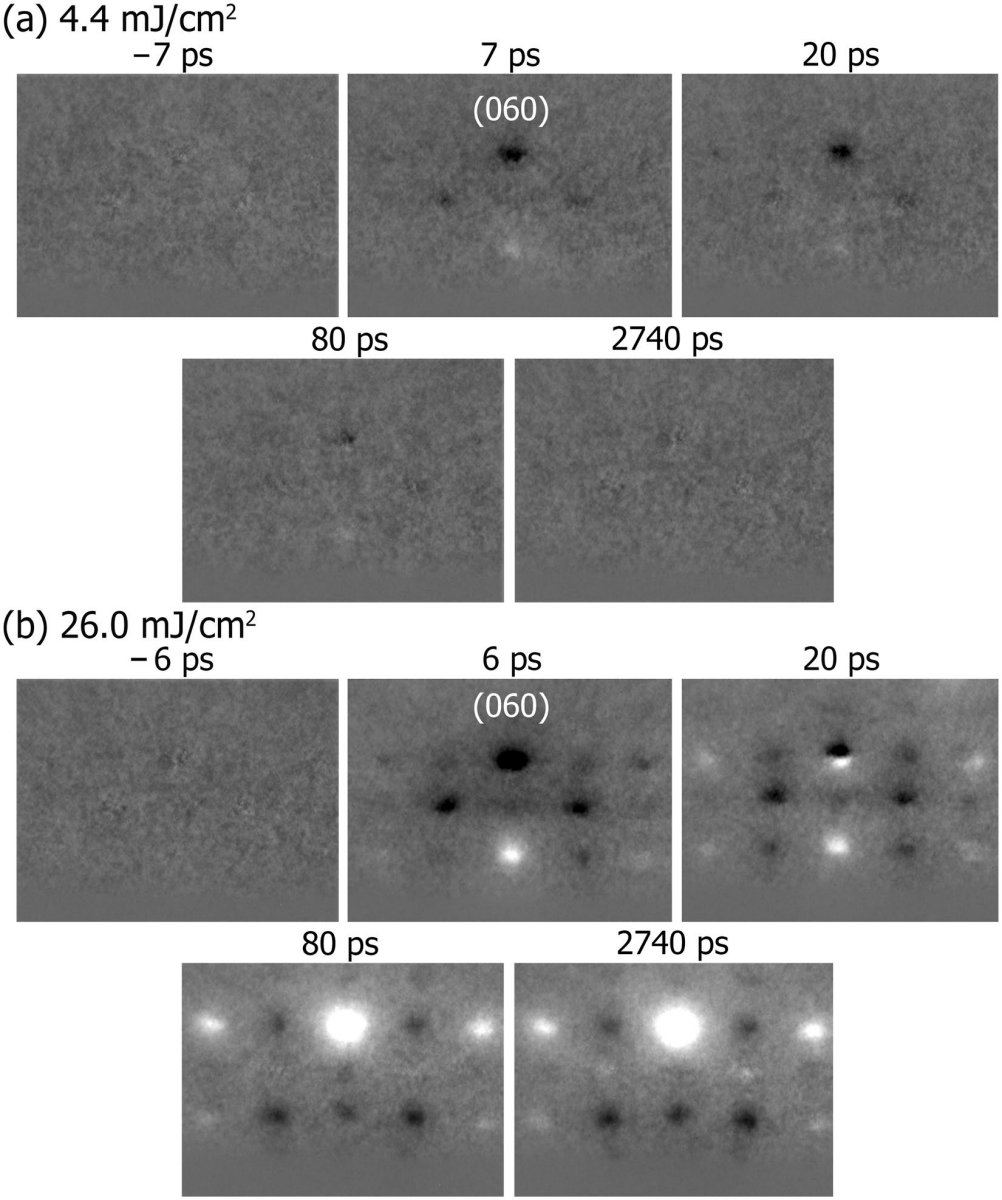
Electron diffraction patterns acquired at selected times following fs photoexcitation. The apparent fluences used were (**a**) 4.4 mJ/cm^2^ and (**b**) 26.0 mJ/cm^2^, respectively.

Shown in Fig. 2 are the diffraction differences at selected delay times at the lowest (*F*_ex_ = 4.4 mJ/cm^2^) and highest (*F*_ex_ = 26.0 mJ/cm^2^) apparent fluences used, referenced to laser-off, nonexcited ED frames. The wavelength was 1030 nm (1.2-eV photons) for an above-gap photoexcitation. It is apparent that a much higher energy density is necessary for the 10-nm film to show sustaining diffraction, thus structural, changes on the nanosecond (ns) scale. The early intensity decrease of the (060) spot and others is visible with dark contrast, where inversion of the intensity change from the dark to bright contrast is seen for a few Bragg diffractions at the high fluence (Fig. 2b). Additionally, the (060) spot (with others also) exhibits a vertical position shift, as indicated by the adjacent dark and bright intensity differences; such a movement signifies a lattice change along the nanoscale, out-of-plane direction. It should be noted that transient electric field effects^49, 50^ were absent in the current study, as we have experimentally confirmed, at the highest fluence used, no observation of movement of the direct electron beam when it was partially blocked by and partially grazing above the laser-excited specimen. In what follows three categories of the rich structural dynamics observed will be discussed: (i) the ultrafast lattice expansion and strain release derived from the (060) movement, (ii) the in-plane motions of vanadium ions at ultrashort times, and (iii) the transformation to the *R* phase at long times.

### Ultrafast strain release dynamics

**Figure 3:**
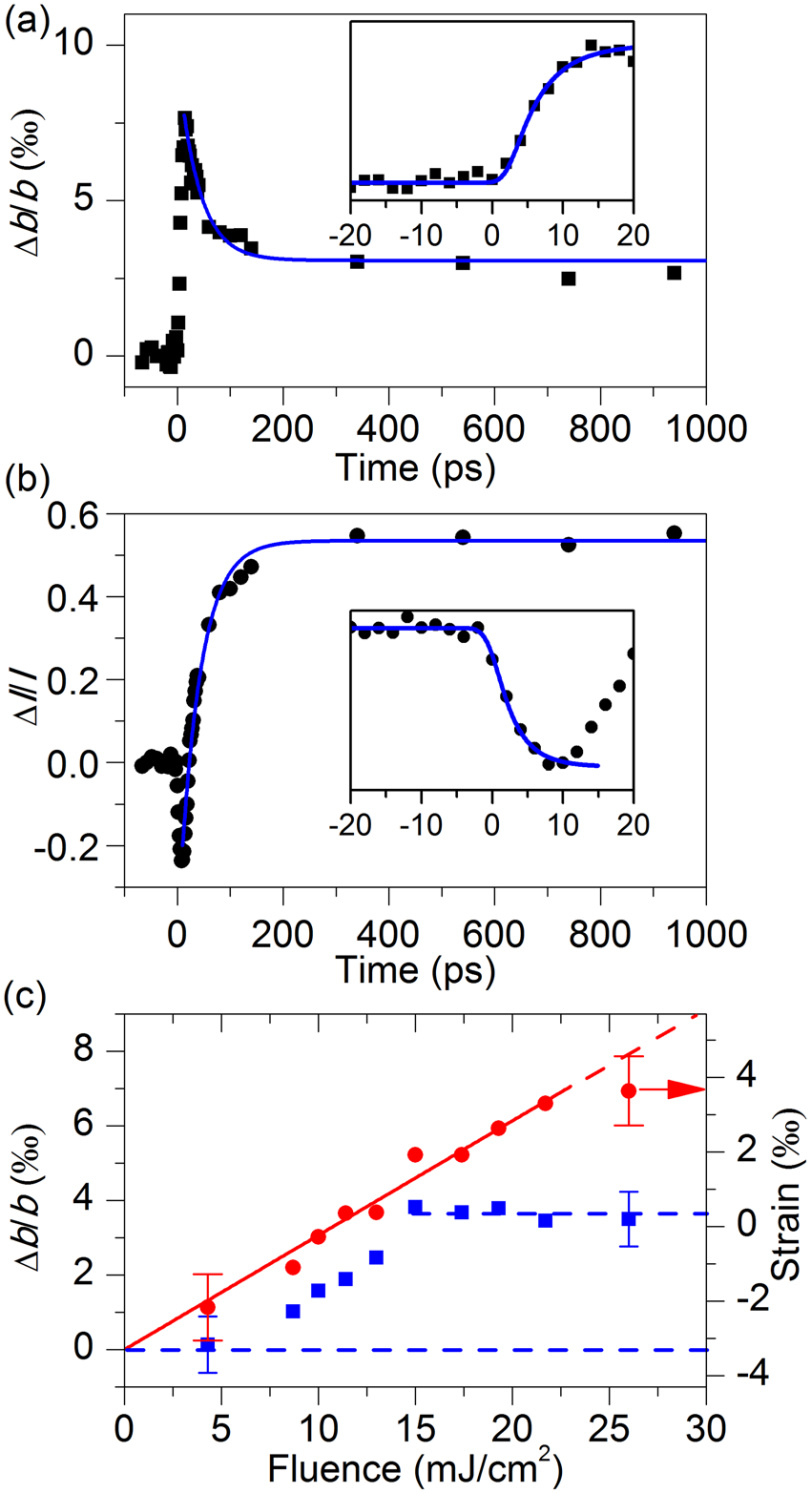
Out-of-plane structural dynamics. (**a**‒**b**) Lattice expansion (black squares) and (060) diffraction intensity (black dots) as a function time at *F*_ex_ = 26.0 mJ/cm^2^. The insets show the early-time dynamics. Blue lines are fits to a single exponential rise or decay function. (**c**) Lattice expansion at 13‒16 ps (red dots) and at 100‒500 ps (blue squares) as a function of *F*_ex_. The corresponding strain relative to the bulk value of ${b}_{{M}_{1}}$ is given on the right. The red arrow indicates the strain associated with the bulk value of *b*_*R*_. The standard deviations calculated from 20 experimental scans are shown for the lowest and highest *F*_ex_ used.

By fitting the vertical profile along the center streak of an ED pattern, the position and intensity of the (060) spot at different times were obtained. An ultrafast lattice expansion in ${b}_{{M}_{1}}$ is seen, which reaches a peak value at > 10 ps with a time constant of *τ*_fast_ = 4.6 ps (Fig. 3a, inset). This out-of-plane expansion in the nanometer dimension is reduced to a sustaining value with a time constant of *τ*_slow_ ≈ 46 ps. Concurrently, the (060) intensity also exhibits a time-dependent response with similar time constants of *τ*_fast_ and *τ*_slow_, first a drop followed by a rise to an enhanced intensity value that remains at long times (Fig. 3b). These observations signify lattice motions of all ions along the ${b}_{{M}_{1}}$ axis (including oxygen for the unit-cell expansion) however in a non-direct-path fashion (causing stepwise intensity changes) on the ps time scales, following the injection of an energy impulse into the ultrathin material.

It is noted that the peak value of the lattice expansion at *t* = 13‒16 ps is linearly proportional to *F*_ex_, except that a saturation appears at the highest apparent fluence used (Fig. 3c, red). The plateau value of 0.7% expansion means *b*_*t*=12ps_ = 4.554 Å, which is incidentally almost the same as the bulk value *b*_*R*_ of the *R* phase^51^. Such a result signifies a complete release of strain at ultrashort times in a highly excited state, from a compressed structure at equilibrium (−0.31% relative to the *M*_1_ bulk) to the supposed final structure of *R* along the noncorrelated *b* axis, even though the multi-stage ionic motions are still ongoing and the *R* phase is yet to be reached. Given the absorption coefficient at 1.2 eV^52^, photoexcitation of the VO_2_ specimen is nearly uniform across the 10-nm film thickness. Thus, the observed structural expansion is reasonably the result of lattice anharmonicity in a photoexcited state and is less relevant to a soundwave propagation or diffusion process^53^.

However, at long times, the largest sustainable lattice expansion in a *c-*sapphire-supported ultrathin film is found close to that for strain-free *M*_1_, or about −0.34% from the *R* bulk, for *F*_ex_ ≥ 15.0 mJ/cm^2^ (Fig. 3c, blue). Moreover, no long-term lattice expansion is seen at *F*_ex_ < 5 mJ/cm^2^, whose excitation threshold behavior coincides with those reported in previous time-resolved studies^26–29, 34, 35, 37, 38^. These results, as well as the long-time dynamics discussed below, suggest that the photoinjected energy is redistributed among multiple components of the structural dynamics, including the overshooting and then reduced amount of strain release as well as the intracell ionic motions along different directions (see below). We argue that this may be one of the reasons why an energy density much higher than the enthalpy change (translated to be ~5 mJ/cm^2^ with 1 to 2-eV photons^35^) is needed to cause the complete transition from *M*_1_ to *R* by photoexcitation, even though an onset threshold close to the thermodynamic requirement has often been seen for ultrafast initiation of the structural phase change in bulk and thicker-film specimens^28, 35^.

### Intracell motions of vanadium ions

**Figure 4:**
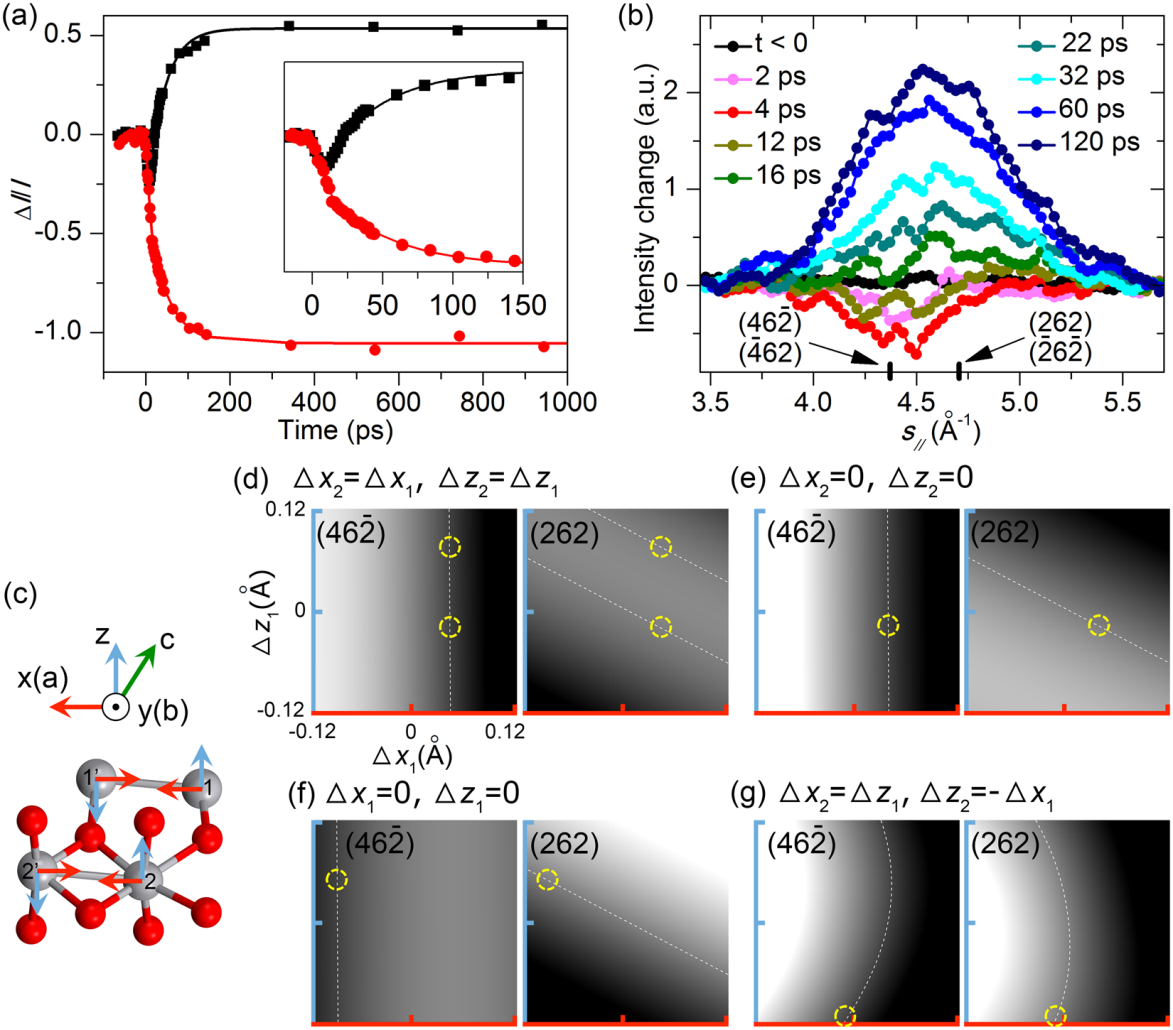
Time-resolved diffraction changes and intracell motions of vanadium ions. (**a**) Intensity change of the (060) (black) and *R*-forbidden spots (red) as a function of time. The solid lines are fits to a single exponential rise or decay function. Inset: Early-time dynamics. (**b**) Intensity profiles across the $(46\bar{2})/(\bar{4}62)$ and $(262)/(\bar{2}6\bar{2})$ diffractions at selected early times, referenced to laser-off, nonexcited frames. (**c**) Intracell motions of vanadium ions in the ${a}_{{M}_{1}}-{c}_{{M}_{1}}$ (i.e., *x*‒*z*) plane considered. The ions of each pair move in opposite directions with the same distance. Simulated intensity changes of $(46\bar{2})/(\bar{4}62)$ (left panels) and $(262)/(\bar{2}6\bar{2})$ (right panels), referenced to the unperturbed state, are shown for (**d**) concerted, same movements for both pairs, (**e**) movements of the first pair only, (**f**) movements of the second pair only, and (**g**) concerted motions involving bond-length change and bond twisting for the two pairs in a unit cell. The white dashed lines denote the parameters that give the experimentally observed intensity changes for the respective diffractions. The yellow dashed circles indicate the potential solutions, taking into account both left and right panels.

Intensity changes on varied time scales are observed for diffractions with nonzero ${h}_{{M}_{1}}$ and ${l}_{{M}_{1}}$ indices. For the *R*-forbidden (i.e., odd-${h}_{{M}_{1}}$) spots, their intensities decrease with an effective time constant of *τ*_slow_ ≈ 46 ps (excluding the early-stage 12 ps), which is the same as that of the (060) intensity rise in the second stage (Fig. 4a). However, an ultrafast intensity drop is specifically seen for $(46\bar{2})/(\bar{4}62)$ within the instrumental response time while the intensity of $(262)/(\bar{2}6\bar{2})$ remains unchanged; afterward, both sets of diffractions show prominent intensity increase on the time scale of *τ*_slow_ (Fig. 4b and Supplementary Fig. S5). Such multi-stage diffraction evolution is qualitatively consistent with the earlier UED studies^34, 35, 37, 38^, although we will point out the distinct differences found here in a strained ultrathin specimen.

To unravel the structural dynamics of the photoinduced phase transition, simulations of diffraction intensities were conducted based on the kinematic scattering theory,

$$I\propto {|\sum _{j}{f}_{j}^{(e)}(s)\cdot \exp (-2\pi i\vec{s}\cdot {\vec{r}}_{j})|}^{2},$$

where ${f}_{j}^{(e)}(s)$ is the atomic scattering factor of the *j*-th atom as a function of *s*, the momentum transfer $s=|\vec{s}|=(4\pi /\lambda )\cdot \sin (\theta /2)$, *λ* = 0.0698 Å is the wavelength of the probe electrons, *θ* is the total scattering angle, and ${\vec{r}}_{j}=({x}_{j}+{\rm{\Delta }}{x}_{j},{y}_{j}+{\rm{\Delta }}{y}_{j},{z}_{j}+{\rm{\Delta }}{z}_{j})$ is the *j*-th atom’s position with the transient movement within a unit cell. For the observed ultrafast dynamics within the first 3 ps (during which the strain release dynamics and ionic motions along ${b}_{{M}_{1}}$ are still underdeveloped), we first examined a few scenarios of concerted vanadium-ion movements in the ${a}_{{M}_{1}}-{c}_{{M}_{1}}$ (i.e., *x*‒*z*) plane in a unit cell, which, for the two ions of a given pair, are in opposite directions but with the same distance (Fig. 4c, red and light blue arrows). Potential solutions were sought to match with the observed intensity decrease of $(46\bar{2})/(\bar{4}62)$ by 24% and no change of $(262)/(\bar{2}6\bar{2})$, including the cases for (1) concerted, same movements for both V‒V pairs in an *M*_1_ unit cell, whose model may share a similar picture as the previous observations of simultaneous bond dilation^34, 37^ (Fig. 4d); (2) movements of only the first pair with the second being stationary (Fig. 4e); (3) movements of only the second pair with the first being stationary (Fig. 4f); and (4) concerted movements with the two pairs switching the amounts of their bond-length change and twisting motion, whose model originates from the structural comparison between *M*_1_ and *M*_2_^38, 51^ (Fig. 4g). However, the theoretical solutions found in the first, second and fourth models are counterintuitive, either positive Δ*x*_1_ in Fig. 4d‒e which signifies contraction of the V‒V dimers, or highly negative Δ*z*_1_ in Fig. 4g which leads to even more tilt for the first pair instead of a straighter alignment. The solution in Fig. 4f seems physically plausible but indicates highly non-uniform ionic motions—large negative Δ*x*_2_ and moderately positive Δ*z*_2_ which means nearly equal bond length with reduced tilt for the second V‒V chain, without modification of the first chain.

Intriguingly, the potential solution in Fig. 4f can also produce the bond-breaking feature^34, 37^ and similar relative intensity changes seen in Fig. 2C at 1 ps of ref. 37; or lead to an intermediate structure containing pairs with different extents of dimerization and tilt and also give resembling relative intensity changes seen in Fig. 4a at 1 ps of ref. 38. However, for a later time at ~20 ps (when the release of strain matures), simulations of the ED pattern changes considering the reaction paths from *M*_1_ to homogeneous bond-breaking, to *M*_2_, or to *R* (Supplementary Fig. S6) fail to produce a result that matches well with the experimentally observed differences (Fig. 2). Taken together, these findings suggest that consideration of a certain structure (bond-breaking, *M*_2_, or *R*) for all unit cells as the intermediate state may not be suitable, at least in the present case using a strained ultrathin specimen. This is in contrast with previous studies^34, 37, 38^.

We note the large position changes of vanadium ions in a unit cell with little adjustments of oxygen when comparing the *M*_1_, *M*_2_, and *R* structures (Supplementary Fig. S7). With the various degrees of freedom possibly involved on the multidimensional energy landscape, it is highly probable that under the influence of photoexcited carriers with excess above-gap energy, the bonded vanadium ions generally move away from each other due to the dynamical nature of the V‒V dimers^18–20^ but also exhibit hot motions in other directions. Small differences in the local chemical environments and the involvement of strain^54^ (such as that along ${b}_{{M}_{1}}$ here), as well as higher photon energy above the band gap, likely cause additional non-uniform (hence non-concerted) motions of vanadium ions in the photoexcited material, which may further influence the nucleation of transformed domains at later times. We believe that this picture provides a connection between the photoinduced and thermally-induced phase transitions of VO_2_, where the transformation proceeds in a mesoscopic, inhomogeneous fashion under the influence of local parameters such as strain and defects^33^.

### Incomplete conversion to the tetragonal structure at long times

**Figure 5:**
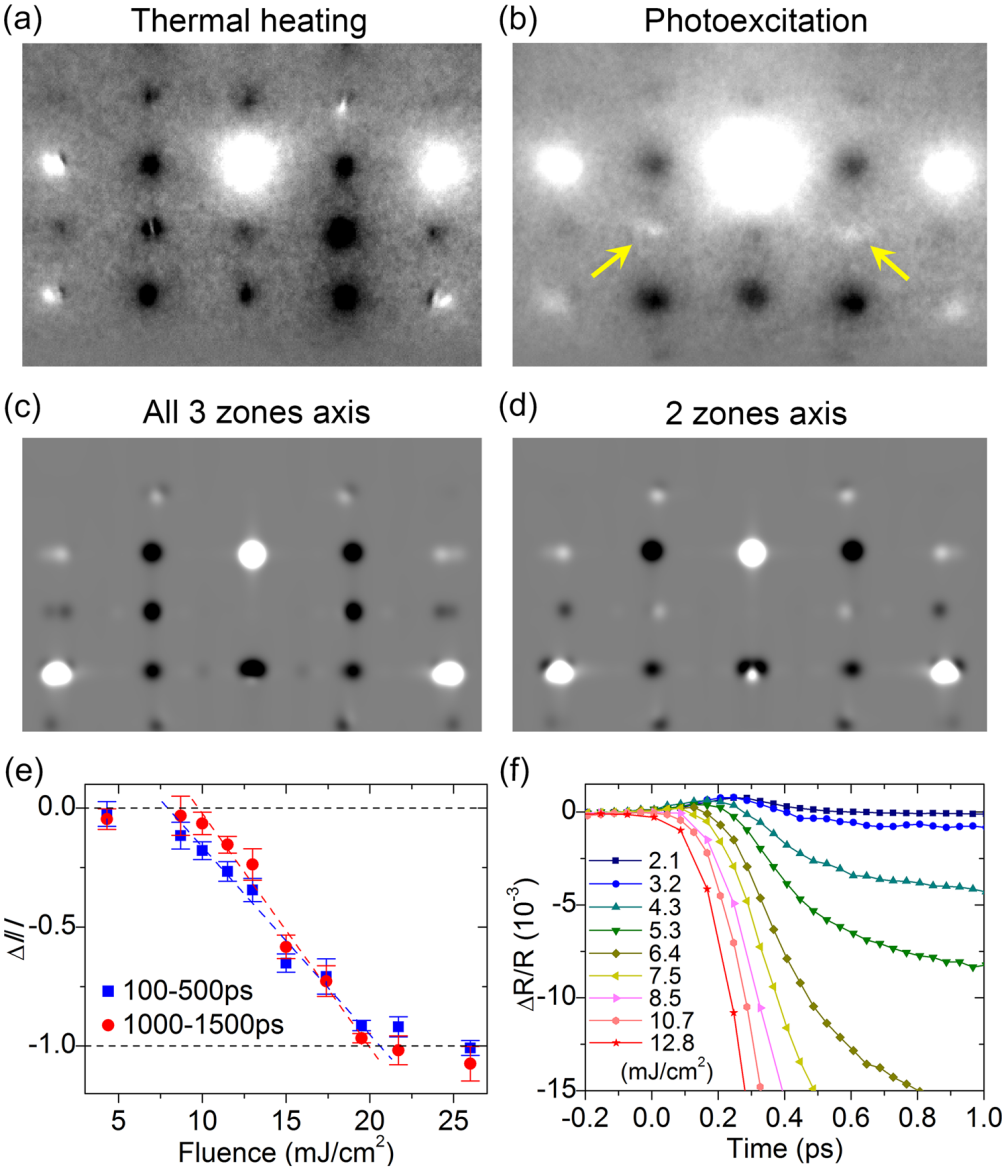
Phase transformation of strained ultrathin VO_2_ and its fluence dependence. (**a**‒**b**) Experimentally observed diffraction differences across the phase transition, induced by thermal heating and by photons, respectively. The main deviation is indicated by arrows. (**c**‒**d**) Simulated diffraction differences between the *M*_1_ and *R* phases, including all three zone axes and only two of the three, respectively. (**e**) Intensity change of the *R*-forbidden spots as a function of the apparent fluence used, for the specified temporal regions. The error bars are the standard deviations calculated from 10‒20 scans. (**f**) Femtosecond transient reflectivity at a probe wavelength of 515 nm for various fluences.

Shown in Fig. 5a‒b are the observed ED differences across the phase transition of VO_2_ using, respectively, thermal heating from 295 to 370 K and photoexcitation at 26.0 mJ/cm^2^ between negative and 2.74-ns times. The apparent deviation between the two patterns contradicts the usual assumption that the photoinduced conversion from *M*_1_ to *R* would complete at long times, at least for high fluences. To investigate the cause, theoretical ED simulations were conducted considering the 6-fold epitaxial relationship and possible combinations of the diffractions from the three zone axes (Fig. 1e‒f, black dashed lines and arrows). The agreement between Fig. 5a,c is satisfactory, which is expected for complete conversion of all crystalline grains from all three zones. However, to account for the slight bright contrast seen on the middle row (spots indicated by arrows in Fig. 5b), at least the pattern from the $\pm (2{c}_{{M}_{1}}^{\ast }-{a}_{{M}_{1}}^{\ast })$ direction, or essentially the diffraction difference from the *R*-forbidden ${(\bar{1}52)}_{{M}_{1}}/{(15\bar{2})}_{{M}_{1}}$ spots, must be excluded. A better agreement is then resulted (Fig. 5b,d).

The exclusion of contribution from a specific zone axis indicates the presence of untransformed crystalline grains on the ns time scale even after all prior fast dynamics have concluded. Thus, such evidence gives strong support for inhibition of the structural transition by surface stress and persistence of those domains in a superheated state for extended times^23, 44^. This result may not be surprising, as signs for an inhomogeneous, impeded phase change have appeared in a former study using time-resolved x-ray diffraction microscopy with a sub-ns temporal resolution at a relatively high excitation fluence^53^. It is also intriguing that the $\pm (2{c}_{{M}_{1}}^{\ast }-{a}_{{M}_{1}}^{\ast })$ direction is the ${c}_{{M}_{1}}$ axis, which exhibits a greater tensile strain compared to that in other directions. This shows the potential of materials engineering to control a phase transformation with different stimuli including strain^55^. Here, we note that inhibition of the structural transformation is observed in the photoexcitation experiments where only the supported ultrathin film is photoheated on a cool, unexcited substrate, but not in the thermodynamic measurements where the whole specimen is heated uniformly. In addition, on the atomic scale, it is reasonable that the interfacial structures are not fully rotationally equivalent for the three zone axes due to the lattice mismatch, the growth process, and contact and overlaying of crystalline domains as seen in Fig. 1g.

For the other two zone axes, the *R*-forbidden $(161)/(\bar{1}6\bar{1})/(26\bar{1})/(\bar{2}61)$ spots lose their intensities completely on sub-ns and ns times at ≥ 20 mJ/cm^2^. We note that this saturation threshold value obtained from long-time structural dynamics matches with that reported using photoinduced transient reflectivity measurements for ultrafast femtosecond phonon oscillations (Fig. 5e)^11^. Such consistency leads us to consider that, without the inhibition by surface stress, a complete photoinduced phase transition of VO_2_ at long times (both electronically and structurally) would require 4 to 5 times of the energy density needed by the thermal phase transition, if 1 to 2-eV photons are used in specimens with strain and/or connected crystalline domains including bulk. In contrast, the initiation threshold is again seen at a lower value of approximately 5 to 7 mJ/cm^2^ where in our transient reflectivity experiments the positive peak begins to be overtaken by the large negative decrease (Fig. 5e‒f), which is consistent with previous reports^11, 29^.

## Discussion

Based on the observed strain release, ultrafast intracell, and long-time structural dynamics as well as the threshold behavior, it is convincing that the excess energy from photoexcitation is channeled into other degrees of freedom, with different time constants, on the multidimensional energy landscape beyond simple breaking of the correlated vanadium chains at least for the current sample condition with strain and connected crystalline domains. This may be analogous to intramolecular vibrational energy redistribution (IVR) observed in photoexcited molecular reactions^56^, although for correlated materials sample conditions such as strain, doping, and stoichiometry can all play a significant role in the redistribution phenomena. Given the converging picture obtained from both dynamics and thermodynamic studies, we believe that the energy redistribution mechanism during the photoinduced phase transition of VO_2_ may not be limited to strained ultrathin specimens but rather broadly applicable to other specimen conditions, especially solid-supported films with domain connectivity. Such a phenomenon has implications for potential use of VO_2_ involving photons or other forms of energy impulse. For example, the higher energy input required for the photoinduced phase transition when strain and/or connected crystalline domains are involved may be advantageous or disadvantageous depending on the applications. Furthermore, it is possible to use photons with low energy for efficient photodoping, to direct the flow of and hence reduce the energy input^38^. Given the present results, it is now crucial to use ultrafast diffraction and spectroscopic methods to study specimens with single-domain crystalline nanobeams and with different chemical doping, in order to examine their impacts on the photoinduced phase transition dynamics and further explore useful control parameters.

In summary, the nature of photoinduced phase transition of VO_2_ is revealed using ultrafast electron diffraction on epitaxially grown ultrathin specimens with moderate strain. The unique specimen conditions allow for investigation of the impacts caused by surface stress on the photoinduced transition dynamics of VO_2_ along different crystallographic directions. Through analysis of the time-dependent changes of Bragg diffractions, the strain release dynamics and ultrafast intracell motions of vanadium ions at early times were unraveled, and the material’s transformation to the presumed final structure was examined up to the nanosecond scale. Strong evidence is found for energy redistribution among the aforementioned structural dynamics in different degrees of freedom and for nonconcerted ionic motions beyond a well-defined reaction intermediate. The presence of the moderate strain and surface stress can lead to inhibition of the structural transition via photoexcitation, within the temporal window observed. We believe that the insights gained through the current study using time-resolved electron diffraction may also be relevant to the photoinduced phase transitions of other correlated materials.

## Methods

### Sample Characterization

Reflection high-energy electron diffraction experiments were performed using a near-parallel beam of 30-keV electrons with a beam diameter of ~85 μm at the specimen. Precision control of the sample position was achieved using a 5-axis high-resolution manipulator (3 translational and 2 rotational degrees of freedom, from McAllister Technical Services) coupled to a cryostat (Janis Research) with an internal heater that enables experiments in the temperature range between < 20 K and 500 K. Temperature was measured with 0.01 K precision at the back of the copper sample holder, and specimen temperatures were independently calibrated with an accuracy of < 1 K by a K-type thermocouple directly attached to the sample surface. Through azimuthal rotations, electron diffractions acquired at different zone axes were used to identify the orientation of VO_2_ films. The transmission-like patterns signify the probing of inner regions beyond the surface layer. The structural phase transition of the specimens was monitored as a function of temperature.

X-ray diffraction (XRD) experiments were conducted using a Rigaku SmartLab diffractometer with the Cu K_α_ radiation (*λ* = 1.54060 Å) and a 5-mm limiting slit. The out-of-plane (*θ*-2*θ*) scan was used to determine the sample orientation along the surface normal direction. To obtain additional confirmation for the epitaxial relationship, two types of in-plane XRD measurements were made using the parallel-beam alignment at fixed grazing incidence angles (*ω*). The first is the *ϕ* scan with the goniometer being rotated azimuthally whereas the detector was fixed at the corresponding 2*θ* of the plane of interest, with *ω* being 0.05° and 0.5° for the VO_2_ film and the substrate, respectively. The second is the 2*θ*-*ϕ* scan where the azimuthal rotations of the sample and the detector were coupled.

### Ultrafast electron diffraction

Time-resolved electron diffraction experiments were carried out using the fundamental output of an amplified laser system (Pharos-SP, Light Conversion) for photoexcitation (at 1030 nm, 1.20 eV, with a full-width-at-half-maximum (FWHM) of 170 fs). The FWHM of the footprint of the excitation beam was ~500 μm following a near-normal impingement at the specimen. Part of the fundamental laser beam passed through two stages of second-harmonic generation; the resulting ultraviolet (257 nm) laser beam were directed and tightly focused onto a 25-μm LaB_6_ emitter tip, embedded in a guard ring, inside the electron gun (Kimball Physics) to generate photoelectron pulses as the probe. At the number of electrons ( < 10^3^) per pulse used, the temporal width at the specimen was below 1 ps^57, 58^.

For structural dynamics, the electron pulses were focused to a 15-μm spot in FWHM, characterized using the knife-edge method. At the incidence of 2.66°, the electron footprint in the reflection geometry was about 300 μm and well within the laser excitation region. The scheme of pulse-front tilt was not employed. The resulting instrumental response time, estimated to be 3 ps given the velocity mismatch between photons and grazing electrons, was experimentally confirmed. At each delay time, both laser-on (photoexcited) and laser-off (nonexcited) frames were acquired in order to capture the actual photoinduced diffraction dynamics and minimize any long-term changes as a result of the laboratory conditions or potential accumulated specimen modifications. Reproducibility of the reported observations was confirmed by repeating the experiments at different apparent fluences multiple times with more than 10 scans and finding the same dynamics in each scan.

To confirm a pure structural origin for the diffraction changes observed, experiments regarding movements of the direct electron beam were conducted, with the VO_2_ specimen surface being parallel to and partially blocking the electron beam path for potential influence caused by Columbic repulsion between different groups of electrons above the photoexcited region^49, 50^. At the highest fluence used (26.0 mJ/cm^2^) no beam movement was observed, thus confirming the absence of photoinduced transient electric field effects. Another piece of supporting evidence is the lack of any background intensity change near the shadow edge at all delay times. Therefore, the lattice expansion associated with the position shifts of the (060) diffraction is real.

### Data and materials availability

All data needed to evaluate the conclusions in the paper are present in the paper and the Supplementary Information. Materials related to this paper may be requested from the authors.

## Electronic supplementary material

Supplementary Information
