# Supplementary material for: Photoinduced Strain Release and Phase Transition Dynamics of Solid-Supported Ultrathin Vanadium Dioxide
Source: Sci Rep. 2017 Aug 30;7:10045. doi: 10.1038/s41598-017-10217-0 (PMC5577108; doi:10.1038/s41598-017-10217-0)
Supplement: Supplementary file 1 — Supplementary Information [file 41598_2017_10217_MOESM1_ESM.pdf]

Supplementary Information

Photoinduced Strain Release and Phase Transition Dynamics of Solid-Supported Ultrathin Vanadium Dioxide

Xing He[1,†], Napat Punpongjareorn[1,†], Weizheng Liang[2], Yuan Lin[2], Chonglin Chen[3], Allan J. Jacobson[1,4], and Ding-Shyue Yang*[,1]

[1] *Department of Chemistry, University of Houston, Houston, Texas 77204, United States*

[2] *State Key Laboratory of Electronic Thin Films and Integrated Devices, University of Electronic Science and Technology of China, Chengdu, 610054, China*

[3] *Department of Physics and Astronomy, University of Texas at San Antonio, San Antonio, Texas 78249, United States*

[4] *Texas Center for Superconductivity, University of Houston, Houston, Texas 77004, United States*

[†] These authors contributed equally.

*To whom correspondence should be address. Email: yang@uh.edu

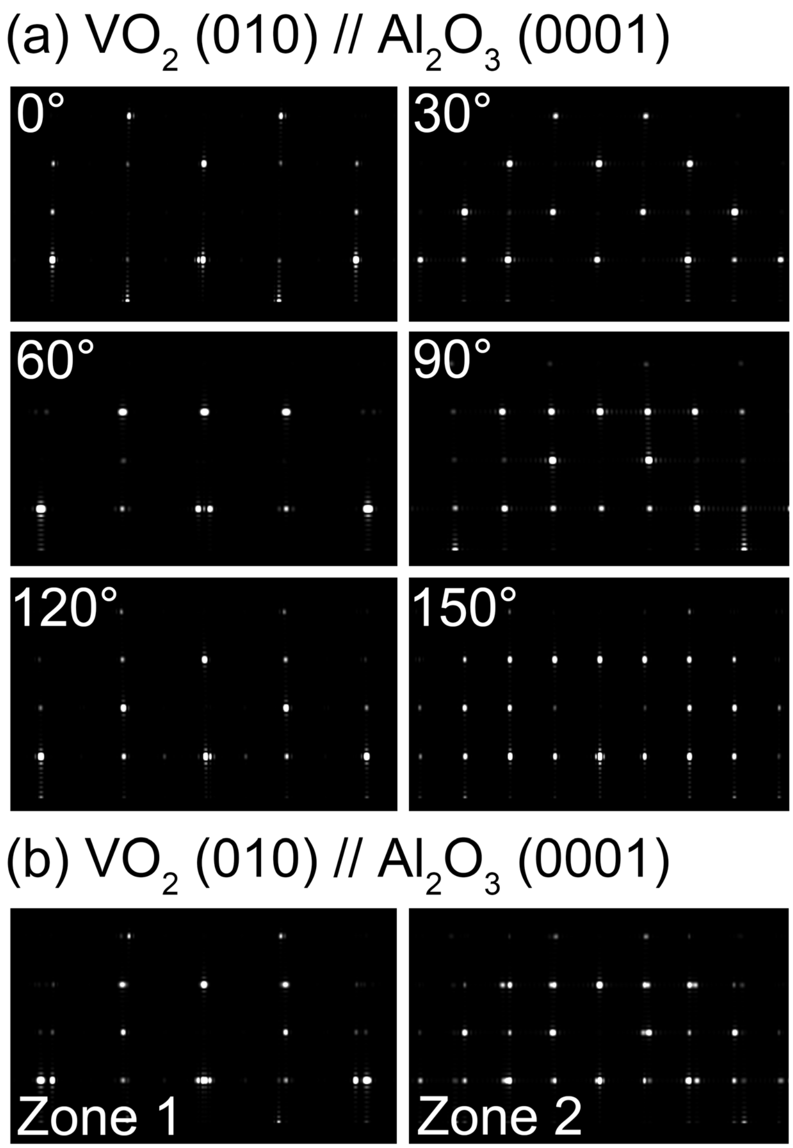

Figure S1. (a) Simulated electron diffraction patterns with selected azimuthal angles, considering the b_{M_1} axis of VO_2 being parallel to the surface-normal direction, Al_2O_3(0001). (b) Anticipated patterns as a result of the 6-fold epitaxial relationship, by summing the patterns at 0°, 60° and 120° for Zone 1 and those at 30°, 90° and 150° for Zone 2.

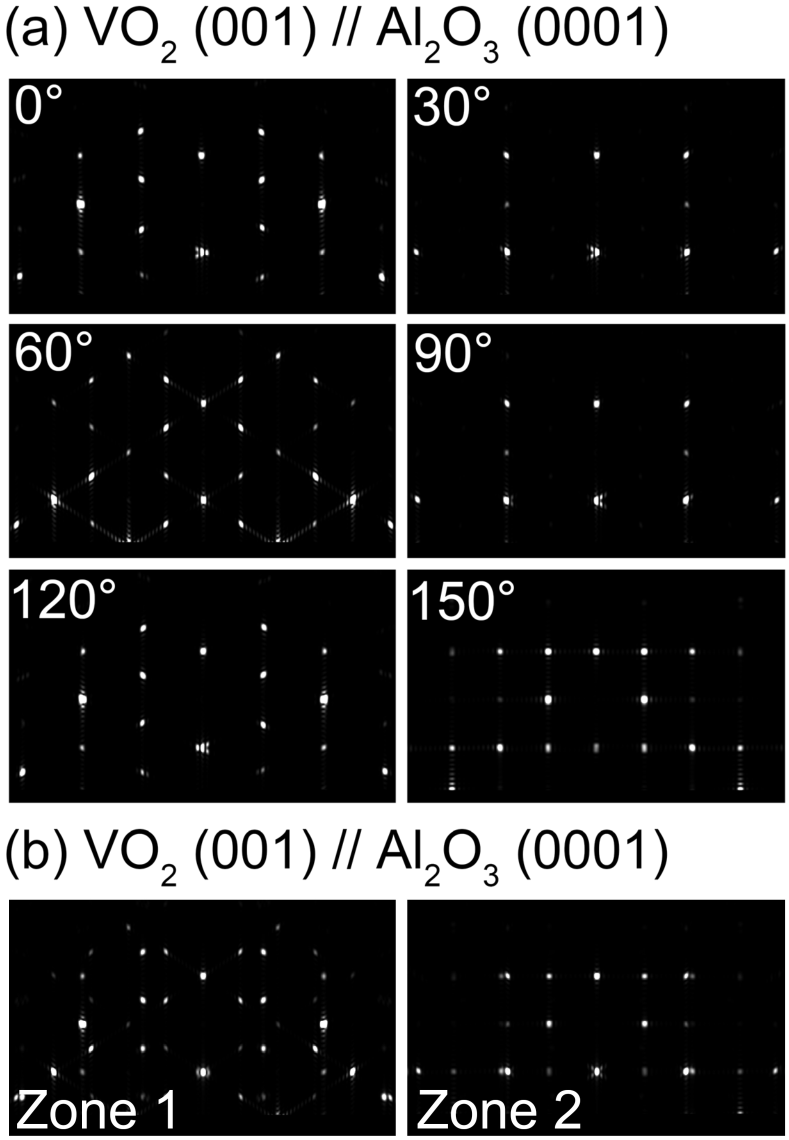

Figure S2. (a) Simulated electron diffraction patterns with selected azimuthal angles, considering the c_{M_1} axis of VO_2 being parallel to the surface-normal direction, Al_2O_3(0001). (b) Anticipated patterns as a result of the 6-fold epitaxial relationship, by summing the patterns at 0°, 60° and 120° for Zone 1 and those at 30°, 90° and 150° for Zone 2. The former pattern was not observed experimentally.

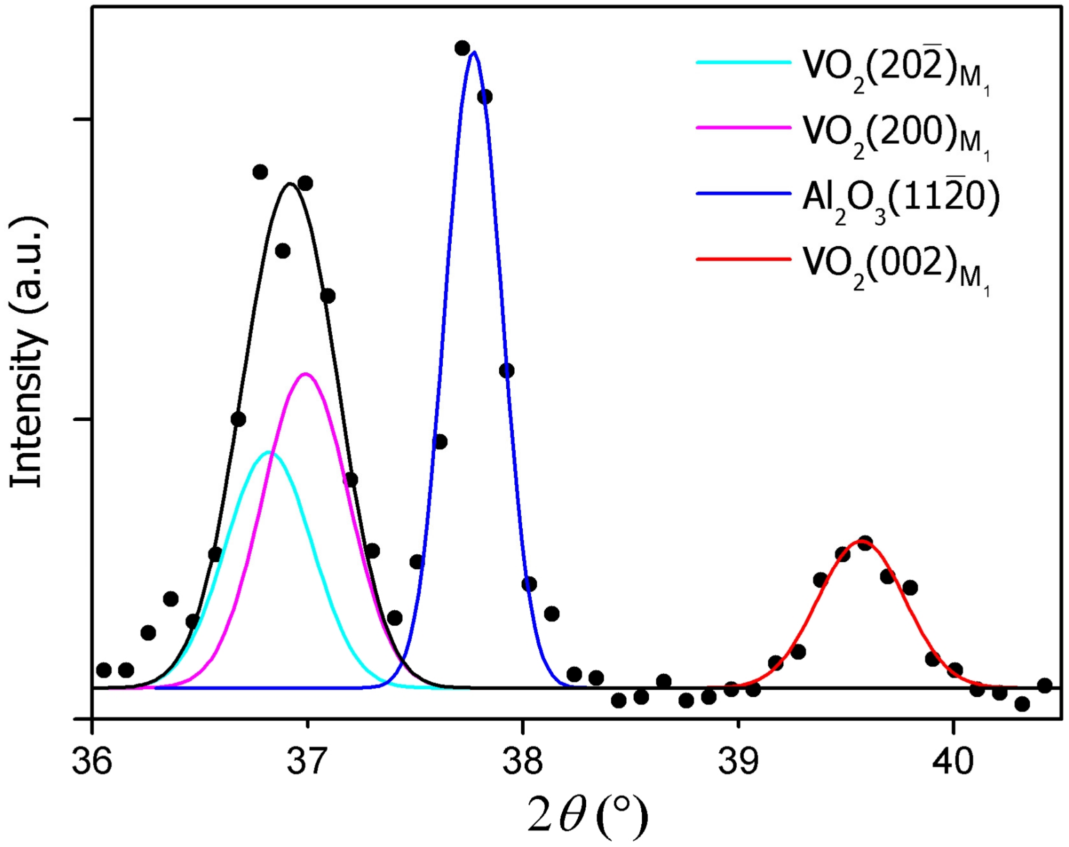

Figure S3. Analysis of in-plane strain using x-ray diffraction data from a 2θ-ϕ scan (black dots). Fits to Gaussian profiles give the 2θ angles for the respective diffractions (solid lines), where a common width is used for all of the VO_2 peaks and the theoretical ratio of the intensities of (200) and $(20\bar{2})$ is considered. The sapphire diffraction peak is used as the reference. From the 2θ angles of (200), $(20\bar{2})$ and (002), the magnitudes of $a^*_{M_1}$, $a^*_{M_1} - c^*_{M_1}$ and $c^*_{M_1}$ were derived using Bragg's law. By trigonometry the angle between $a^*_{M_1}$ and $c^*_{M_1}$ was calculated from multiple scans and an average value of $\beta_{M_1} = 122.59°$ was obtained.

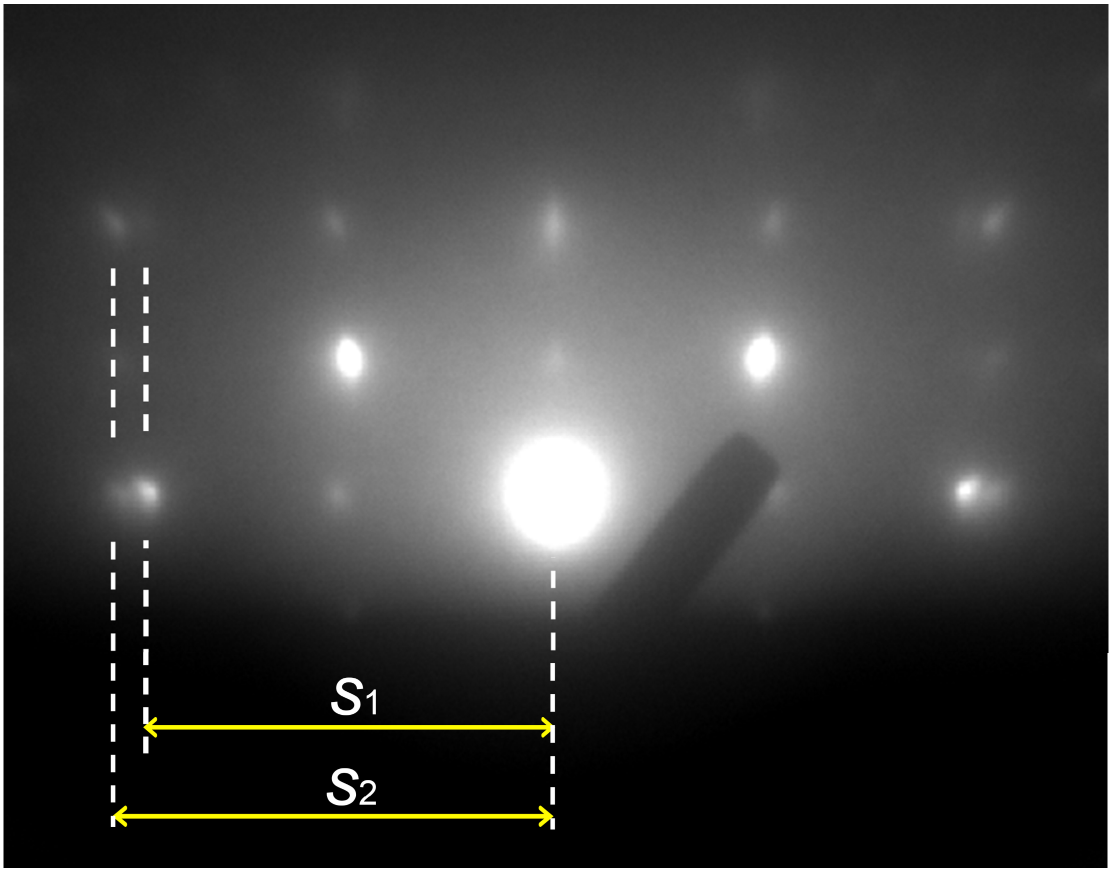

Figure S4. Analysis of in-plane strain using an electron diffraction pattern from Zone 1. The horizontal momentum transfers of s_1 and s_2 correspond to $2 \times \left|2a^*_{M_1} - c^*_{M_1}\right|$ and $2 \times \left|a^*_{M_1} + c^*_{M_1}\right|$, respectively. The difference between the experimentally observed values and the bulk values of 4.370 and 4.710 Å^{-1} gives the in-plane strain. The result obtained is consistent with that of Fig. S3.

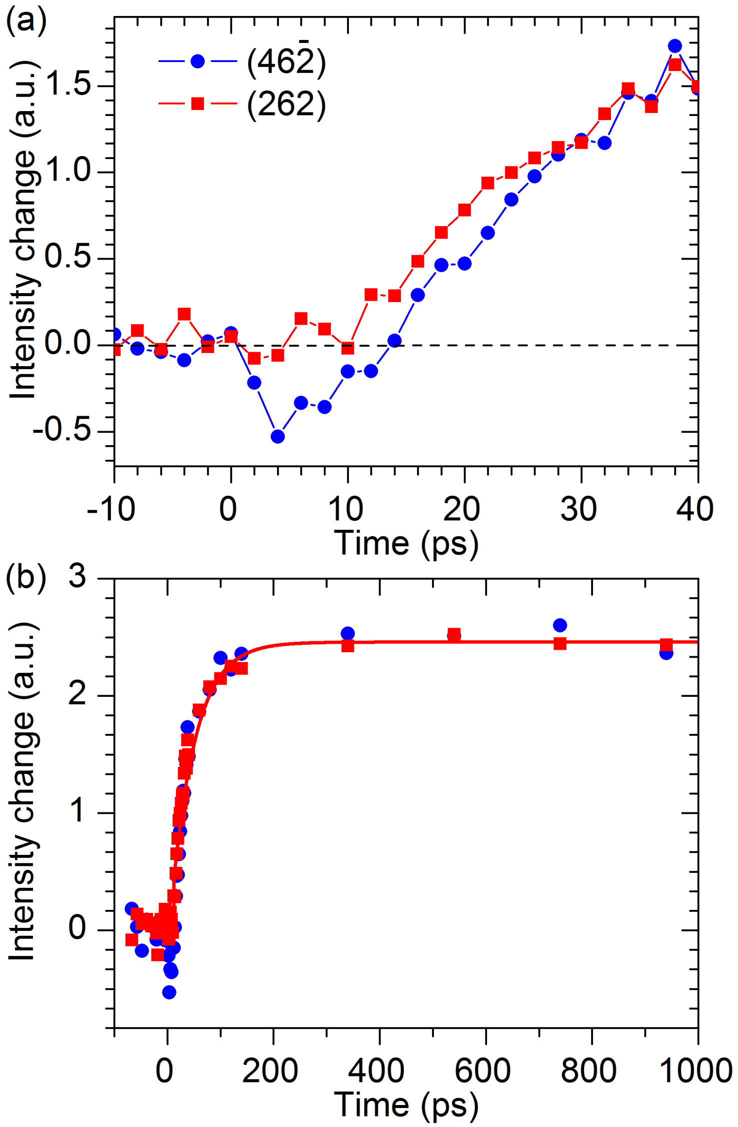

Figure S5. Intensity change of the $(46\bar{2})/(\bar{4}62)$ (blue dots) and $(262)/(\bar{2}6\bar{2})$ (red squares) diffractions at (a) early times and (b) longer times. The ultrafast intensity decrease of $(46\bar{2})/(\bar{4}62)$ takes place within the instrumental response time of 3 ps. The red solid line in (b) is a fit to an exponential rise function with an effective time constant of $\tau_{slow} \approx 46$ ps, excluding the first 12 ps.

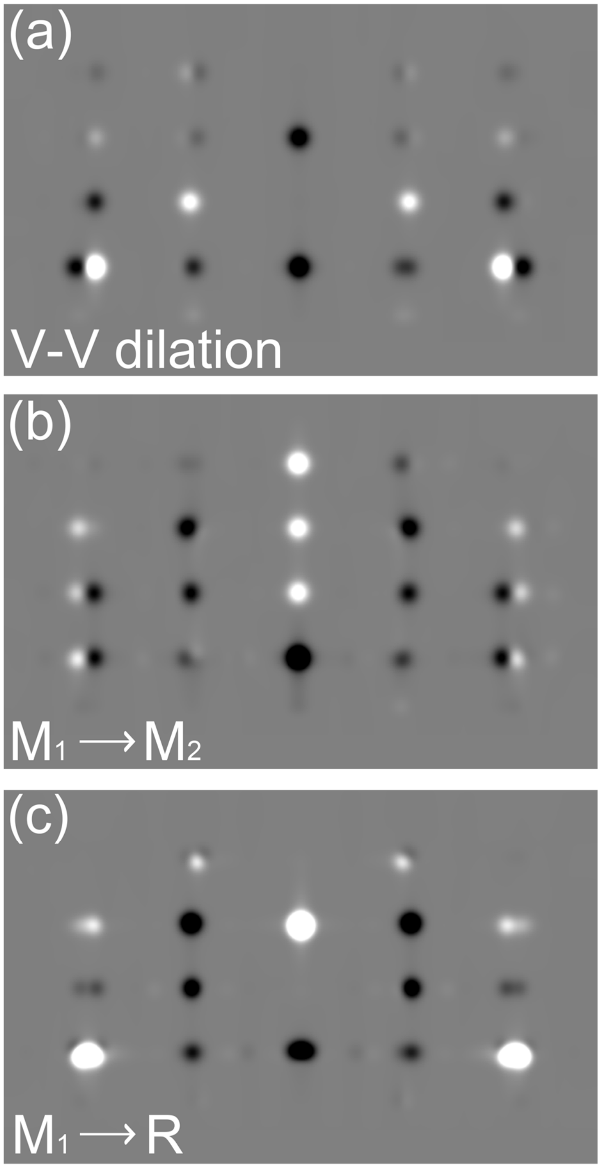

Figure S6. Simulated electron diffraction differences considering the structural transformations of (a) uniform dilation along the V−V bonds by 0.10 Å for each vanadium ion, (b) M_1 to M_2 without the relative lattice strain, and (c) M_1 to R.

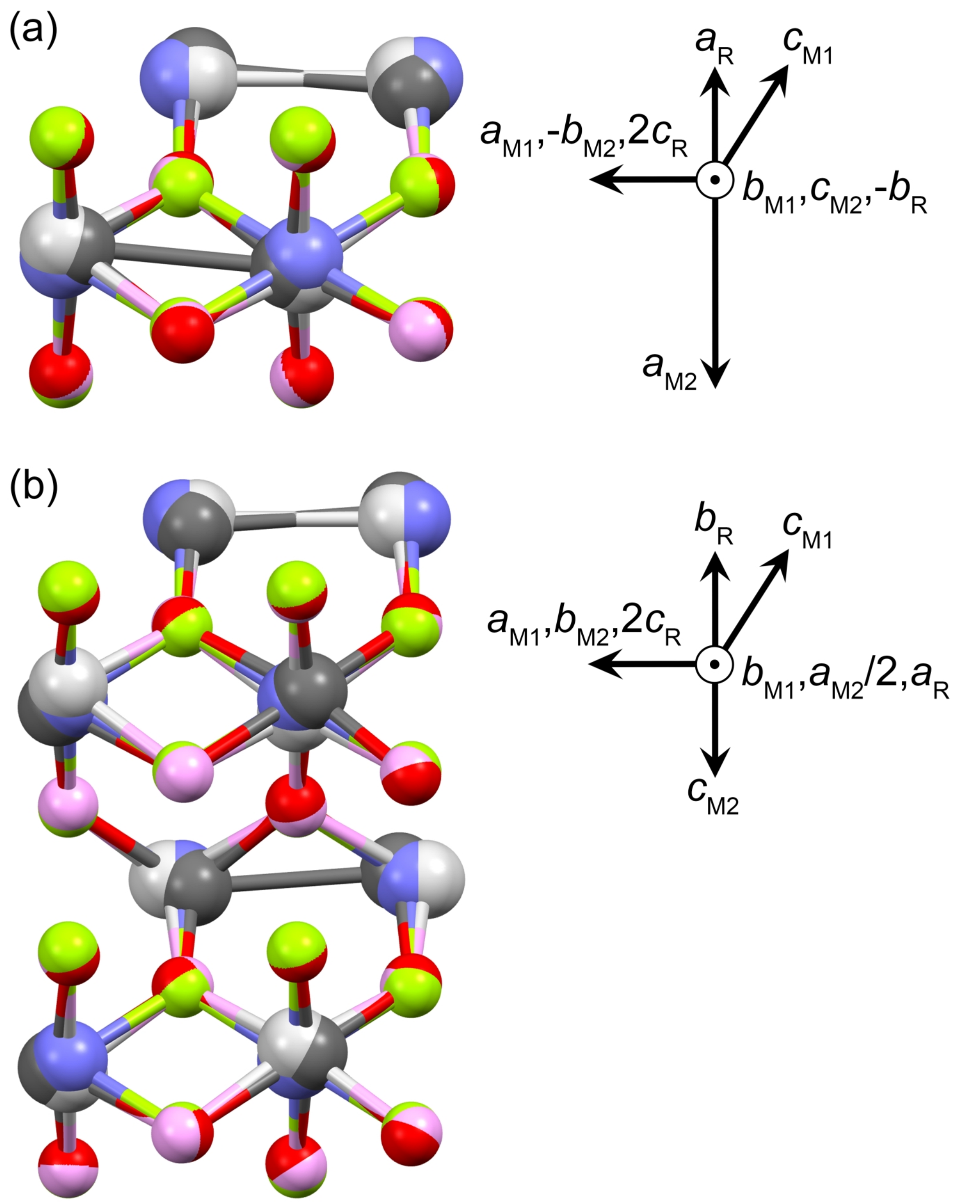

Figure S7. Structural comparison of the M_1 (V in dark gray and O in red)[1], M_2 (V in white and O in pink)[2], and R (V in purple and O in green) phases of VO_2, after unit-cell scaling to exclude the interference of the small lattice strain. The movements of the vanadium ions among the three phases are prominent, whereas the sphere-looking oxygen ions signify only minor position adjustments for the oxygen octahedra. (a) The comparison for a unit cell of M_1 according to the model presented in Figure 8 of Ref. 2 and in Ref. 3. (b) The comparison for a unit cell of M_2 according to the matrix equations of Ref. 2 (with oxygen ions included). The four different ways of V–V pair movements are not the commonly used picture although the matrix equations have been accepted in the literature.

References

1. Longo, J. M. & Kierkegaard, P. A refinement of structure of VO_2. *Acta Chem. Scand.* **24**, 420-426 (1970).
2. Marezio, M., McWhan, B., Dernier, P. D. & Remeika, J. P. Structural aspects of metal-insulator transitions in Cr-doped VO_2. *Phys. Rev. B* **5**, 2541-2551 (1972).
3. Eyert, V. The metal-insulator transitions of VO_2: A band theoretical approach. *Ann. Phys. (Leipzig)* **11**, 650-702 (2002).
